# Repeat photography of Langtang Valley, Nepal (1949–2025): A dataset for monitoring landscape and settlement changes

**DOI:** 10.1016/j.dib.2026.112703

**Published:** 2026-03-23

**Authors:** Marcus Nüsser, Susanne Schmidt, Alexander Klaus

**Affiliations:** a Department of Geography, South Asia Institute (SAI), Heidelberg University, Voßstraße 2/4230, 69115 Heidelberg, Germany; b Heidelberg Center for the Environment (HCE), Heidelberg University, Im Neuenheimer Feld 130.1, 69120 Heidelberg, Germany

**Keywords:** Photographical surveys, Glacier, Settlement changes, Tourism destination, Himalaya, Nepal

## Abstract

This article presents a multi-temporal dataset of historical photographs and replicates from the Langtang Valley, located in the Central Himalaya of Nepal. The collection and collation of historical images from various archives and thematically focused repeat photography surveys enables detailed analysis of landscape, glacier and settlement changes. Characterized by harsh topography and glaciers at higher elevations, the high mountain landscape of Langtang Valley is also known as a prominent destination for mountain tourism. Based on historical landscape and settlement images, rephotographic surveys were conducted in the years 2008, 2012, 2017, 2018, and 2025. Hence, the dataset allows for comparative views before and after the massive 2015 Gorkha Earthquake, which heavily affected the Langtang Valley. The first part of the dataset (A) contains a set of ten examples of bi- and multi-temporal landscape photographs and allows to detect glacier changes, including debris cover, ice cliffs, and disconnection from headwalls. The second part (B) contains a set of twelve examples of bi- and multi-temporal photographs that focuses on structural and functional changes in the two main settlements Langtang village and Kyanjin Gompa, both affected by the 2015 mega-rupture. The imagery taken before and after the earthquake illustrate the rapid reconstruction activities and the massive transformation process, mainly driven by mountain tourism. The dataset can be used by glaciologists, climate scientists, geographers and planning experts in urban development and tourism. It supports a recently submitted article (Klaus et al. 2026, Before and after the 2015 Disaster: Development Trajectories in the Himalayan Riskscape of Langtang from 1949 to 2025, Progress in Disaster Science) with original archival material.

Specifications Table

**Table:** 

Subject	Earth & Environmental Sciences
Specific subject area	The photographical dataset contains multi-temporal imagery to document landscape, glacier and settlement changes in Langtang Valley, Nepal.
Type of data	Image, Map.
Data collection	All available historical photographs were evaluated and selected according to their topical relevance and quality. The original glass plates of the metric photographs (13 cm ×18 cm) taken by Gerhard Moser in 1980 were collected from the archive of the Austrian Alpine Club, Innsbruck and scanned at 600 dpi with a large format scanner. Photographs from the Himalayan collection of Christian Kleinert taken in the 1970s were selected from the archive at South Asia Institute, Heidelberg University, Germany. Printed and digital versions of historical photographs were used to relocate the original viewpoints for repeat photography. During repeat photography surveys, the camera was mounted on a tripod (Gitzo Mountaineer) for careful selection of view angles and frames.
Data source location	Region: Langtang Valley, Rasuwa District Country: Nepal Latitude and longitude for collected data: 28.16°–28.27°N; 85.44°–85.69°E, Elevation: 3400–5000 m a.s.l*.*
Data accessibility	The dataset described in this study are available from the Mendeley repository and can be accessed at: Repository name: Multi-temporal photographic dataset of landscape changes in Langtang Valley, Nepal Data identification number: 10.17632/c2cffxzmm2.1 Direct URL to data: http://dx.doi.org/10.17632/c2cffxzmm2.1 Instructions for accessing these data: The data are online available and downloadable at Mendeley. For peer review only: the data can be downloaded anonymously.
Related research article	Klaus et al. 2026, Before and after the 2015 Disaster: Development Trajectories in the Himalayan Riskscape of Langtang from 1949 to 2025, Progress in Disaster Science (submitted)*.*

## 1 Value of the Data

• This dataset provides a detailed photographic documentation of glacier and human-environmental changes in a central Himalayan valley over a long period from 1949 to 2025.
• The data allows for future monitoring of landscape, glacier and settlement changes providing detailed information of environmental and urbanisation processes in the Langtang Valley since 1949. Possibilities include the integration of high-resolution remote sensing data and unmanned aerial vehicles as well as additional repeat photography surveys to document development at various spatio-temporal scales.
• The multi-temporal documentation of landscapes and glaciers enables geographers, glaciologists and environmental researchers to assess climate-driven changes and the effects of geohazards.
• The multi-temporal documentation of settlements and infrastructure allows regional planners, disaster specialists, tourism experts and heritage conservationists to rely on site-specific visual representations of high spatio-temporal resolution.
• The imagery of two main settlements of Langtang Valley before and after the 2015 earthquake enables to investigate rapid socio-economic transformations, mainly driven by the development of mountain tourism and the dynamics of post-disaster reconstruction.

## 2 Background

High mountain environments are generally characterized by massive and often rapid landscape changes due to climatic conditions and sudden geohazards. At the same time these sensitive regions are often prominent and exposed destinations for tourism, regularly accompanied by urbanisation processes and socioeconomic changes. The Langtang Valley in Nepal is a case in point to demonstrate the intricate relationship between different human-environmental dimensions. Heavily affected by the 2015 Gorkha earthquake, this Himalayan Valley can be characterized as a high mountain riskscape, shaped by geological and tectonic settings, climatic conditions and by rapid urbanisation processes. The photographic dataset comprises historical images and replicates that demonstrate both the magnitude of glacier changes and the dynamics of urbanisation processes before and after the earthquake. Against this background, the dataset collates multi-temporal photographs in high spatio-temporal resolution, visualizing the dynamics of contemporary landscape and settlement changes in a sensitive mountain environment. The photographic collection adds value to the co-submitted article: Klaus et al. 2026, Before and after the 2015 Disaster: Development Trajectories in the Himalayan Riskscape of Langtang from 1949 to 2025, Progress in Disaster Science [1].

## 3 Data Description

**Fig. 1:**
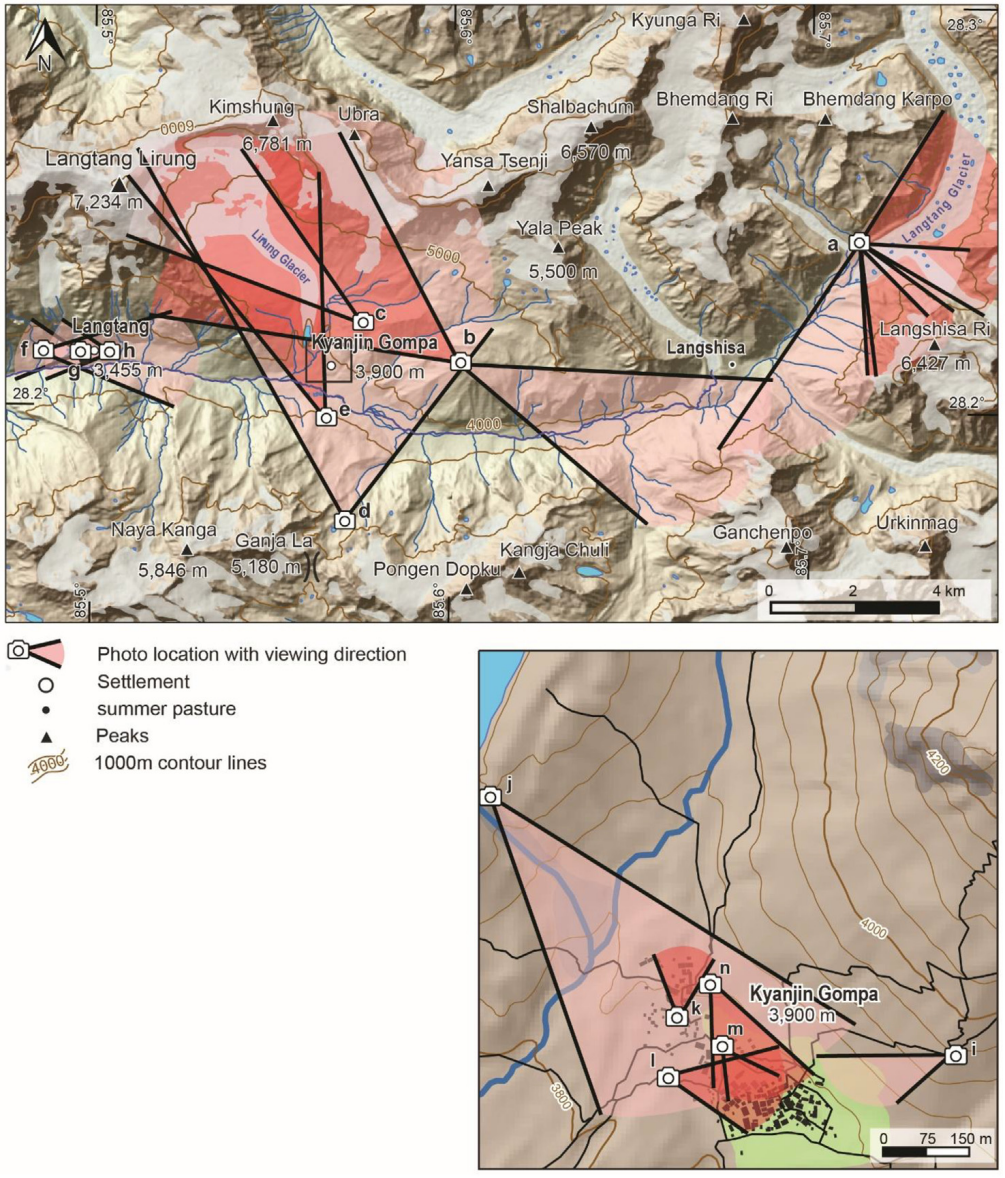
Map of central Langtang Valley with viewpoints and viewing directions. a: Morimoto Base Camp, b: Tsergo Ri, c: Kyanjin Ri, d: Near Ganja La, e: Near Ngegang Kharka, f: Langtang Village West, g: Langtang Memorial, h: Langtang Village East, i: Kyanjin Gompa North, j: Lirung Moraine, k: Kyanjin Gompa West, l: Kyanjin Gompa Southwest, m: Kyanjin Gompa Center, n: Kyanjin Gompa Monastery.

**Fig. 2:**
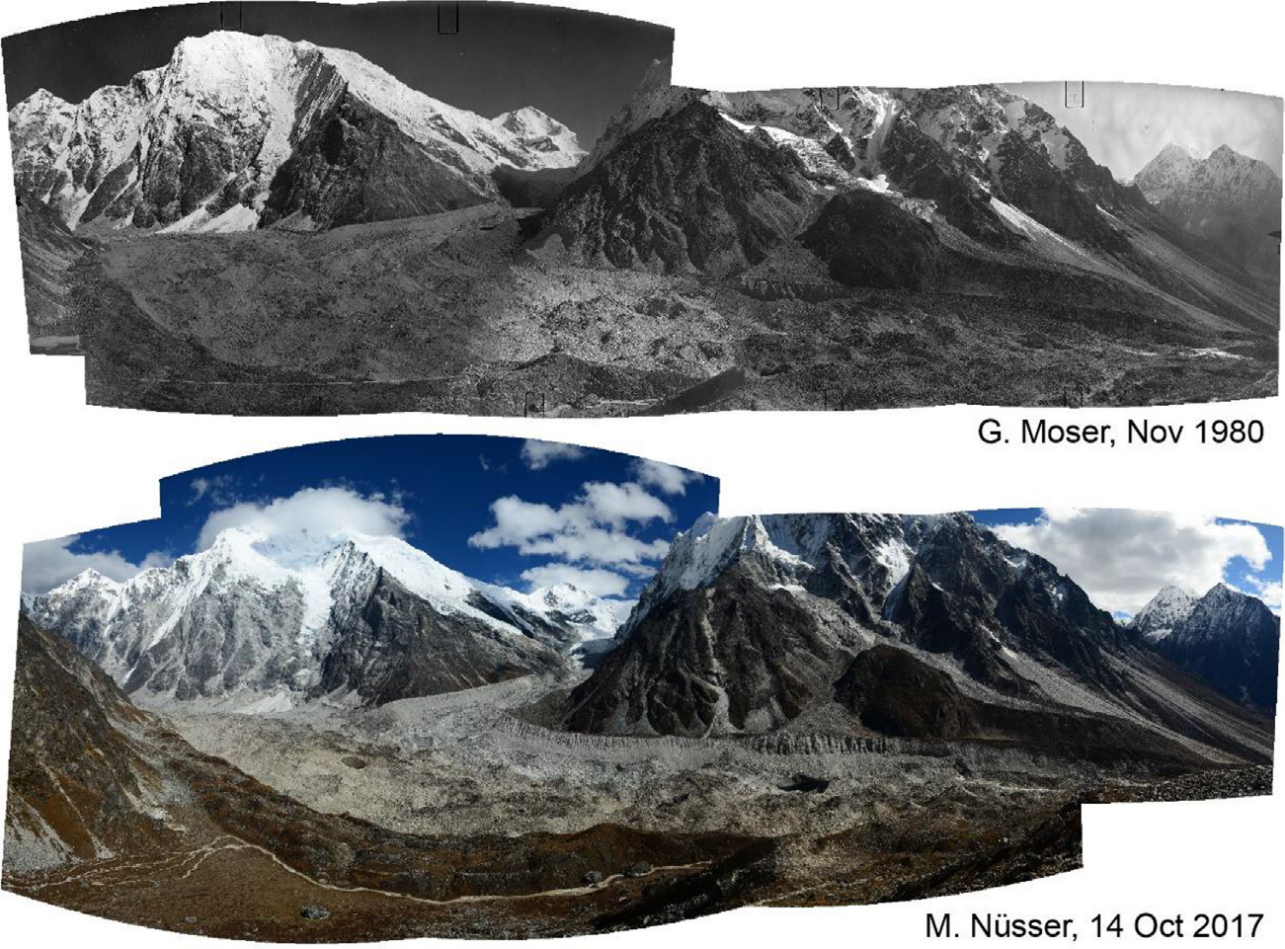
This bi-temporal panorama of matched photographs from 1980 to 2017 shows the lower section of the Langtang Glacier with the confluence of the tributary Nyanang Phu Glacier from Southeast. It is taken from the slope above the orographic right moraine of Langtang Glacier at an elevation of 4725 m (viewpoint a). The viewing direction is towards Southeast with Langshisa Ri (6413 m) in the center, Pemthang Karpo Ri (6830 m) in the upper left corner and Gurkarpo Ri (6889 m) in the central background. The photomosaic provides an overview of the individual pairs of repeat photography (Figs. 3—6), which show more details. The plain area of the Morimoto Base Camp (4600 m) is visible in the lower left corner of the photomosaic from 2017.

**Fig. 3:**
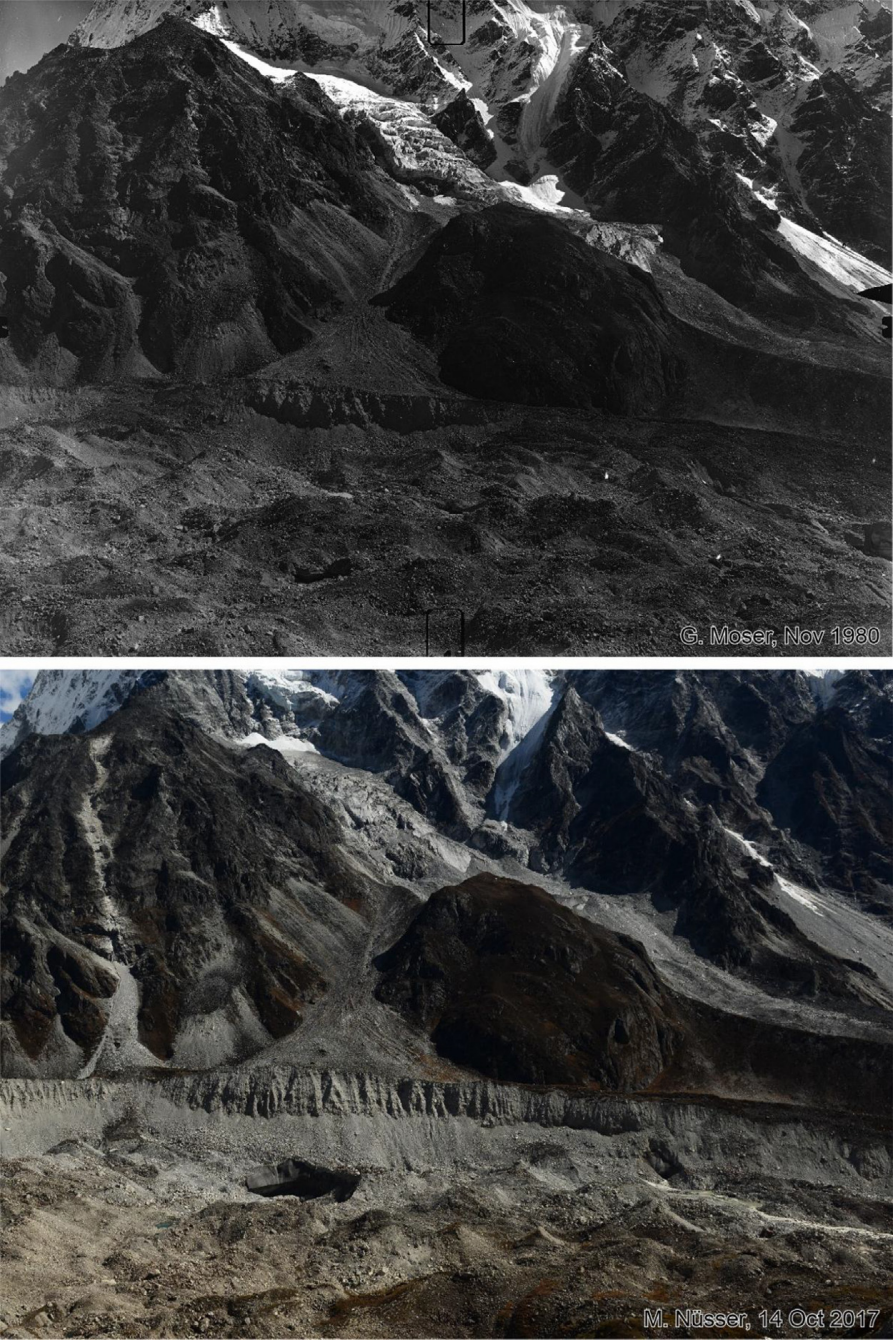
This bi-temporal pair of photographs from 1980 to 2017 shows the lower section and orographic left lateral moraine of Langtang Glacier looking towards south with the lower flank of Langshisa Ri and hanging glaciers, taken from the slope above the orographic right moraine of the glacier at an elevation of 4725 m (viewpoint a). Compared to the situation in 1980, the replicate shows massive downwasting (thinning) of the debris covered glacier in the foreground, the formation of an ice cliff near the lateral moraine and a new outlet of the glacier stream. Some supraglacial vegetation patches can be identified.

**Fig. 4:**
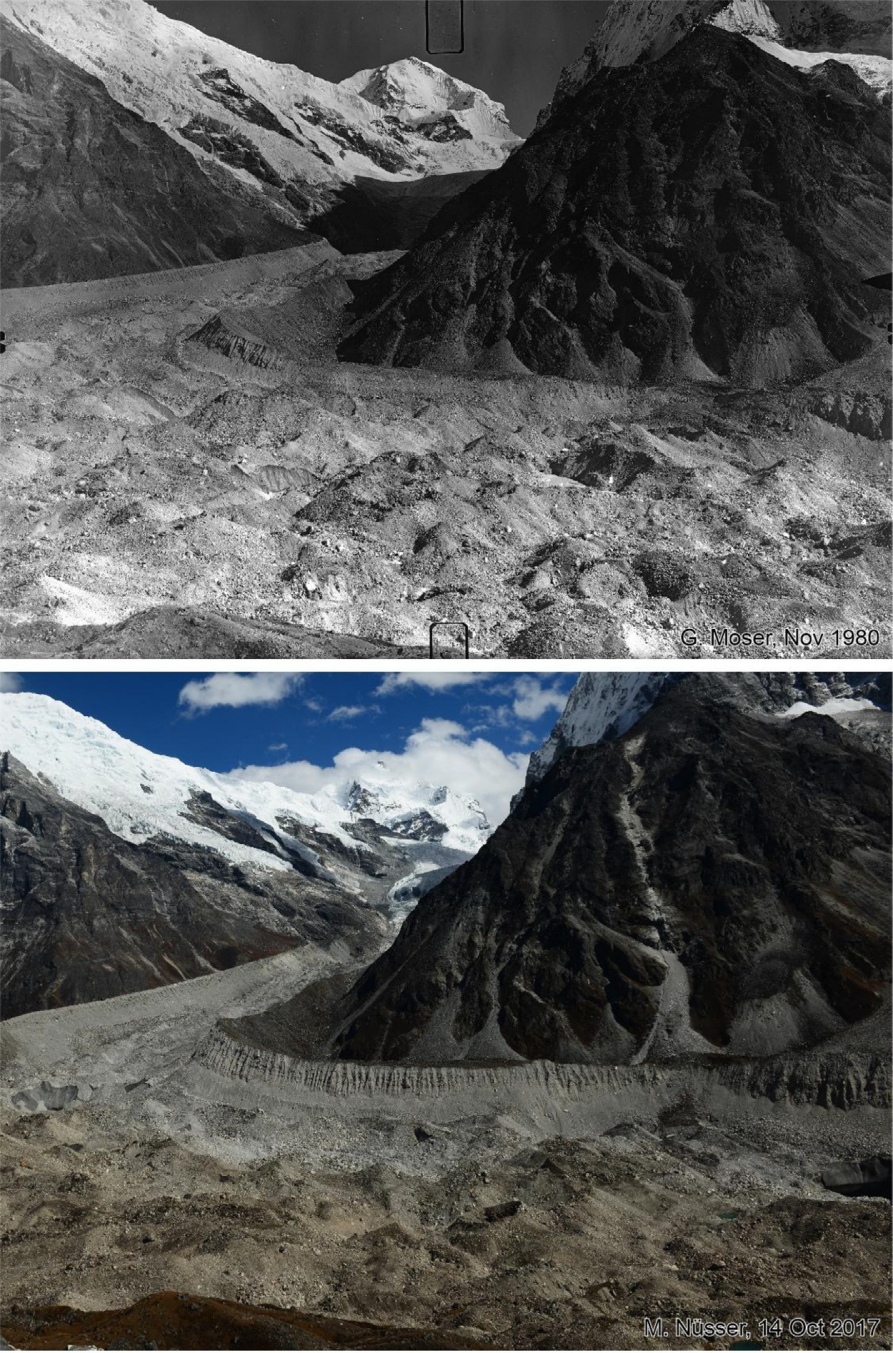
This bi-temporal pair of photographs from 1980 to 2017 shows the confluence of the Langtang Glacier with the tributary Nyanang Phu Glacier. The view is towards southeast from the slope above the orographic right moraine of Langtang Glacier at an elevation of 4725 m (viewpoint a). The view shows the lower flank of Langshisa Ri on the right hand, Gurkarpo Ri (6889 m) in the central background and the western slope of Pemthang Karpo Ri (6830 m) in the upper left corner. Compared to the situation in 1980, the replicate shows massive downwasting (thinning) of the debris covered glacier in the foreground and the formation of a small water pond on the right-hand side. Ice cliffs can be detected near the lateral moraine in the extreme right part of the glacier and at the confluence in the left part of the glacier. While the debris on the surface of the tributary glacier is grey, the main Langtang Glacier has a brown debris cover.

**Fig. 5:**
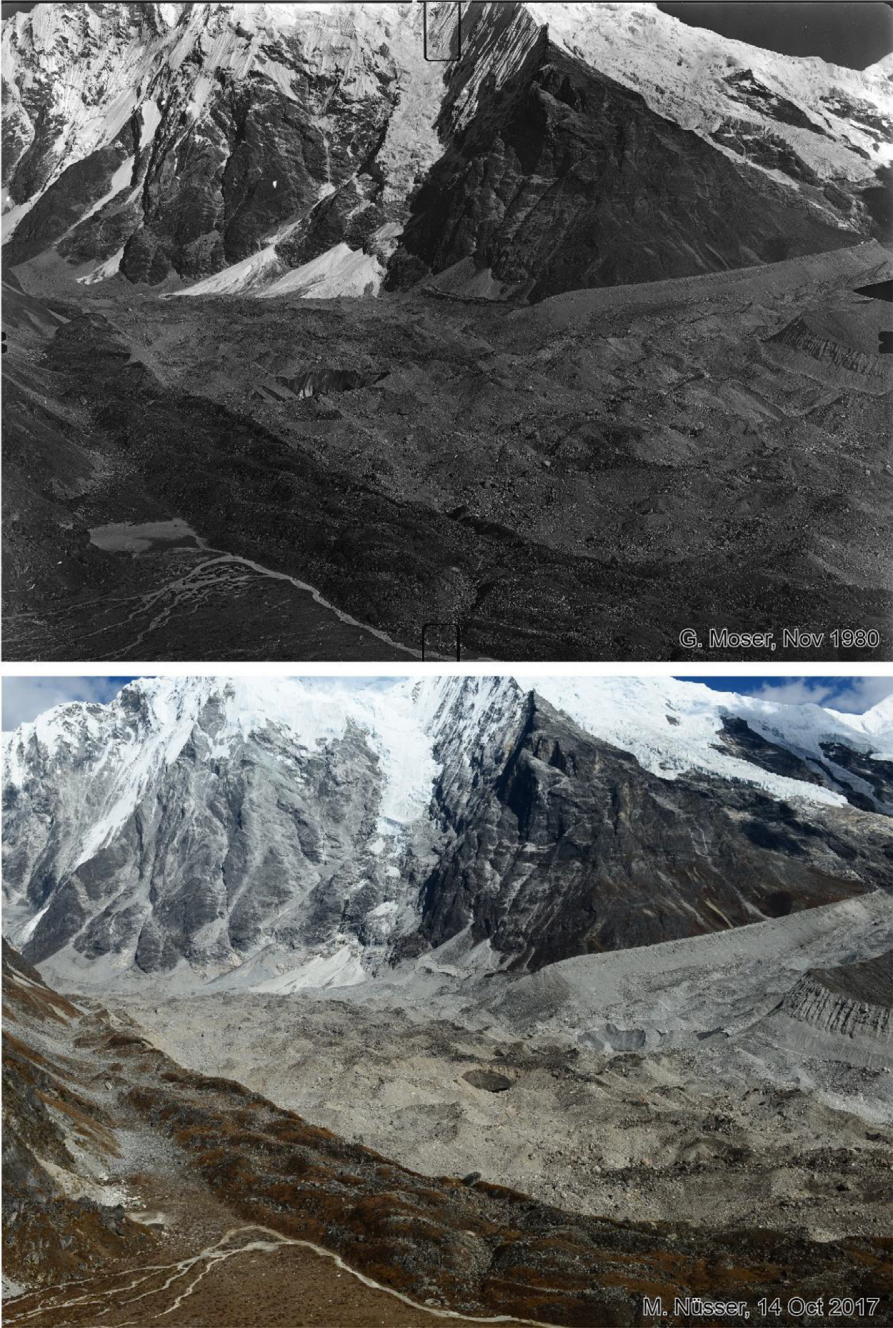
This bi-temporal pair of photographs from 1980 to 2017 shows the confluence of the Langtang Glacier with the tributary Nyanang Phu Glacier. The view is towards southeast with Pemthang Karpo Ri (6830 m) in the background, taken from the slope above the orographic right moraine of Langtang Glacier at an elevation of 4725 m (viewpoint a). Compared to the situation in 1980, the replicate shows massive downwasting (thinning) of the debris covered glacier in the centre and the formation of ice cliffs near the glacier confluence. One ice cliff can already be detected in the photograph from 1980. While the debris on the surface of the tributary glacier is grey, the main Langtang Glacier has a brown debris cover. The lateral moraine remains largely stable with the same individual stones that can be detected in both photographs. The plain area of the Morimoto Base Camp (4600 m) with braided streams in the left foreground also remains largely unchanged.

**Fig. 6:**
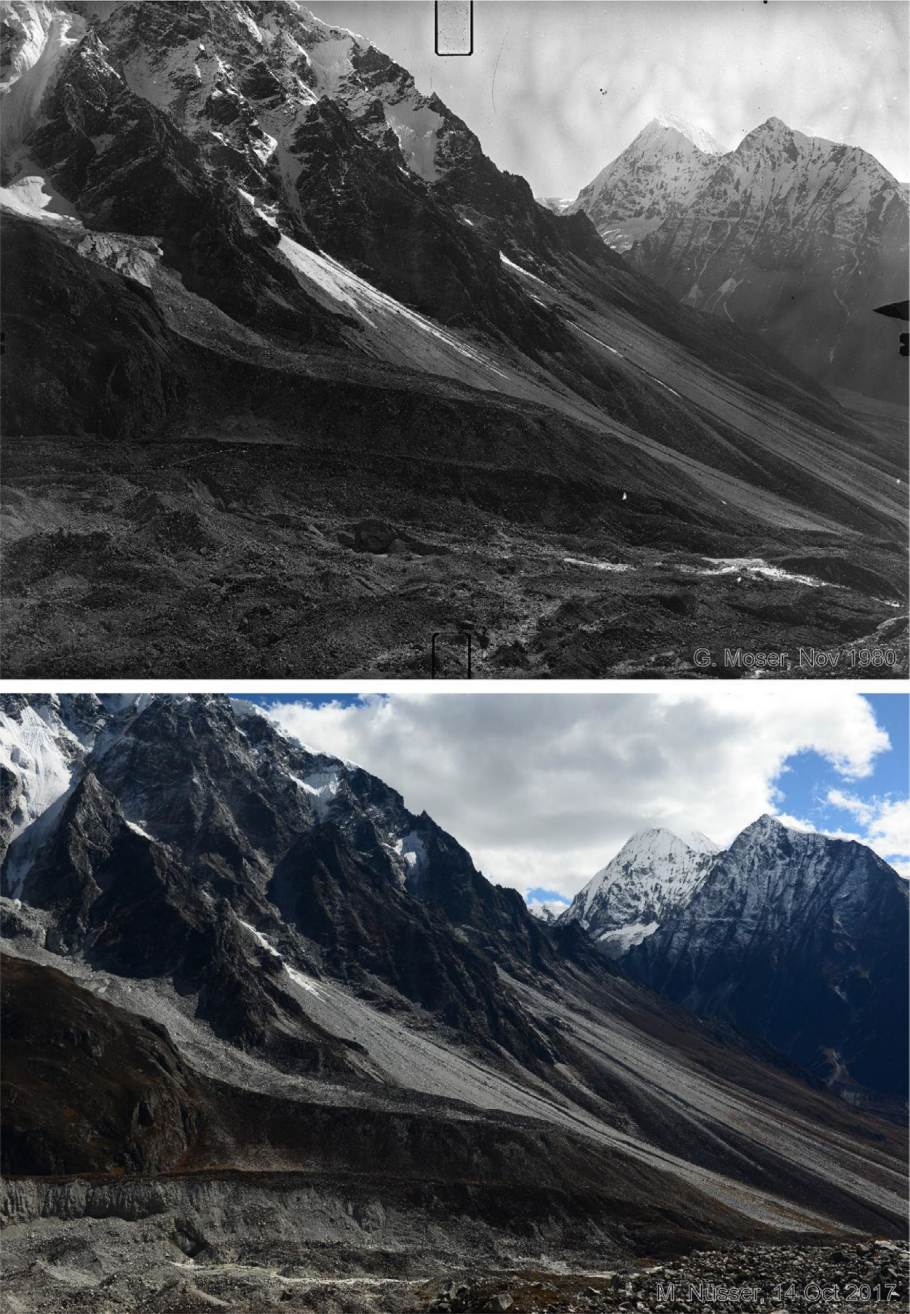
This bi-temporal pair of matched photographs from 1980 to 2017 shows the snout of the Langtang Glacier from the slope above its orographic right moraine at an elevation of 4725 m (viewpoint a). The viewing direction is towards southwest with the lower slopes of Langshisa Ri in the center and Gangchenpo (6387 m) in the right background. Compared to the situation in 1980, the replicate shows massive downwasting (thinning), retreat of the debris covered glacier and a decline of the hanging glaciers, while the periglacial straight slopes and the terminal moraine in the centre remain largely unchanged.

**Fig. 7:**
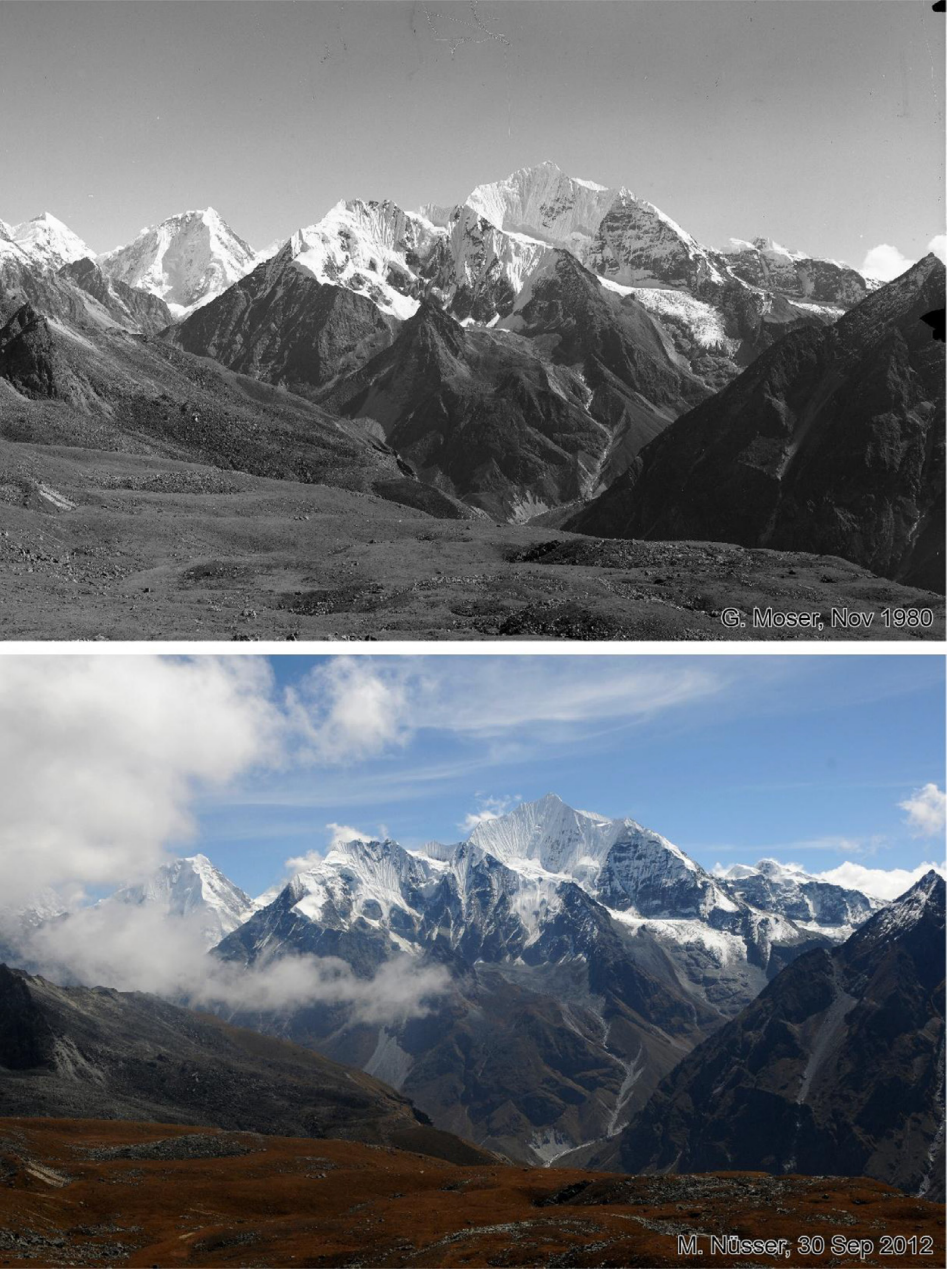
This bi-temporal pair of photographs from 1980 to 2017 shows the mountain massive of Gangchenpo (6387 m) with striking formations of fluted snow on the ice walls. The images are taken from Tsergo Ri (5002 m) (viewpoint b). The glaciers on the north face of Gangchenpo show mass loss and retreat, while the summit plateau of Tsergo Ri in the foreground remains largely unchanged.

**Fig. 8:**
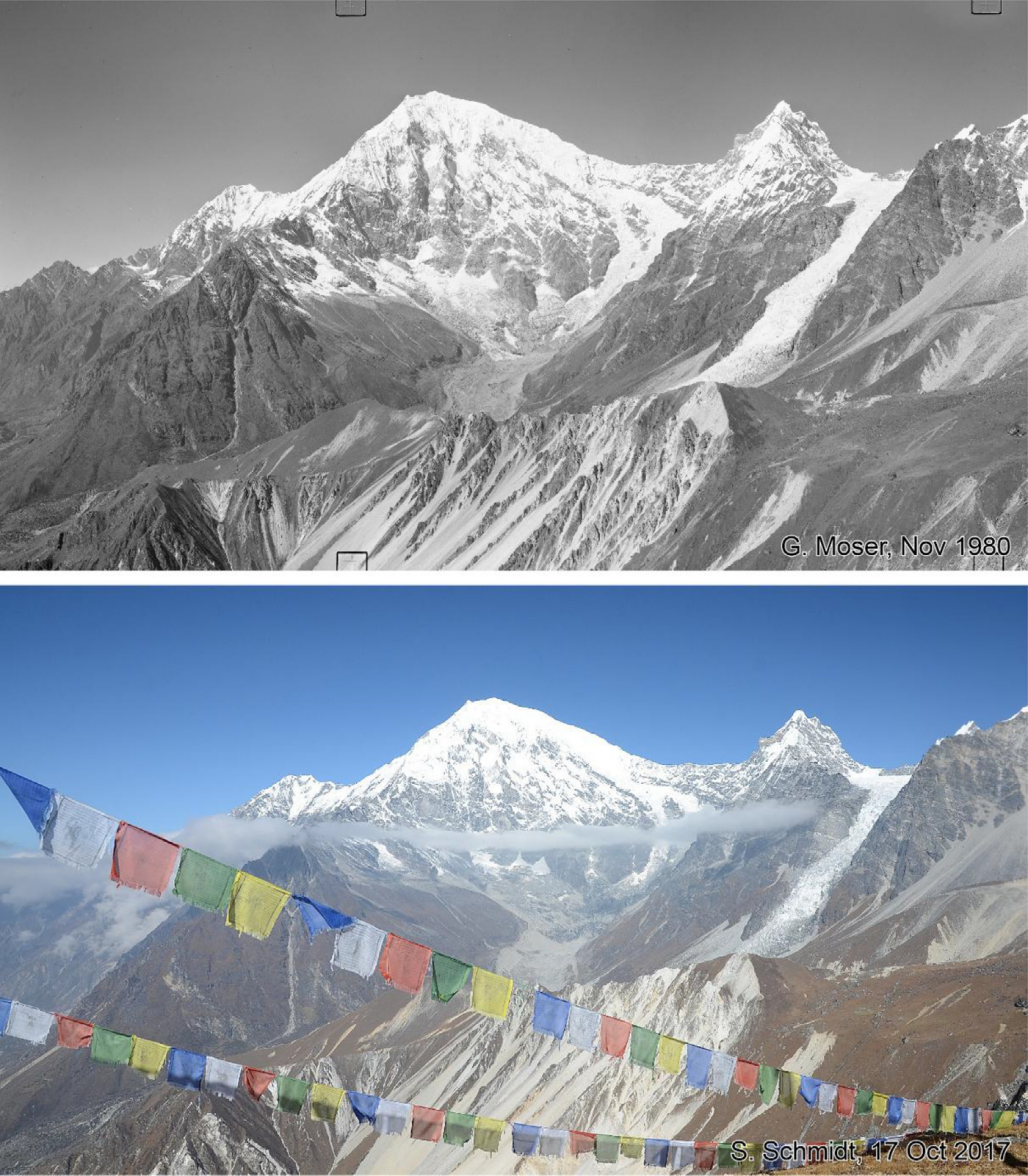
This bi-temporal pair of photographs from 1980 to 2017 shows Langtang Lirung (7234 m) with the Lirung Glacier in the centre and the peak of Kimshung (6686 m) with the clean ice Kimshung Glacier to the right-hand side. The images are taken from Tsergo Ri (5002 m) (viewpoint b). A loss of ice volume in the debris covered part of the Lirung Glacier and the steep Kimshung Glacier can be detected.

**Fig. 9:**
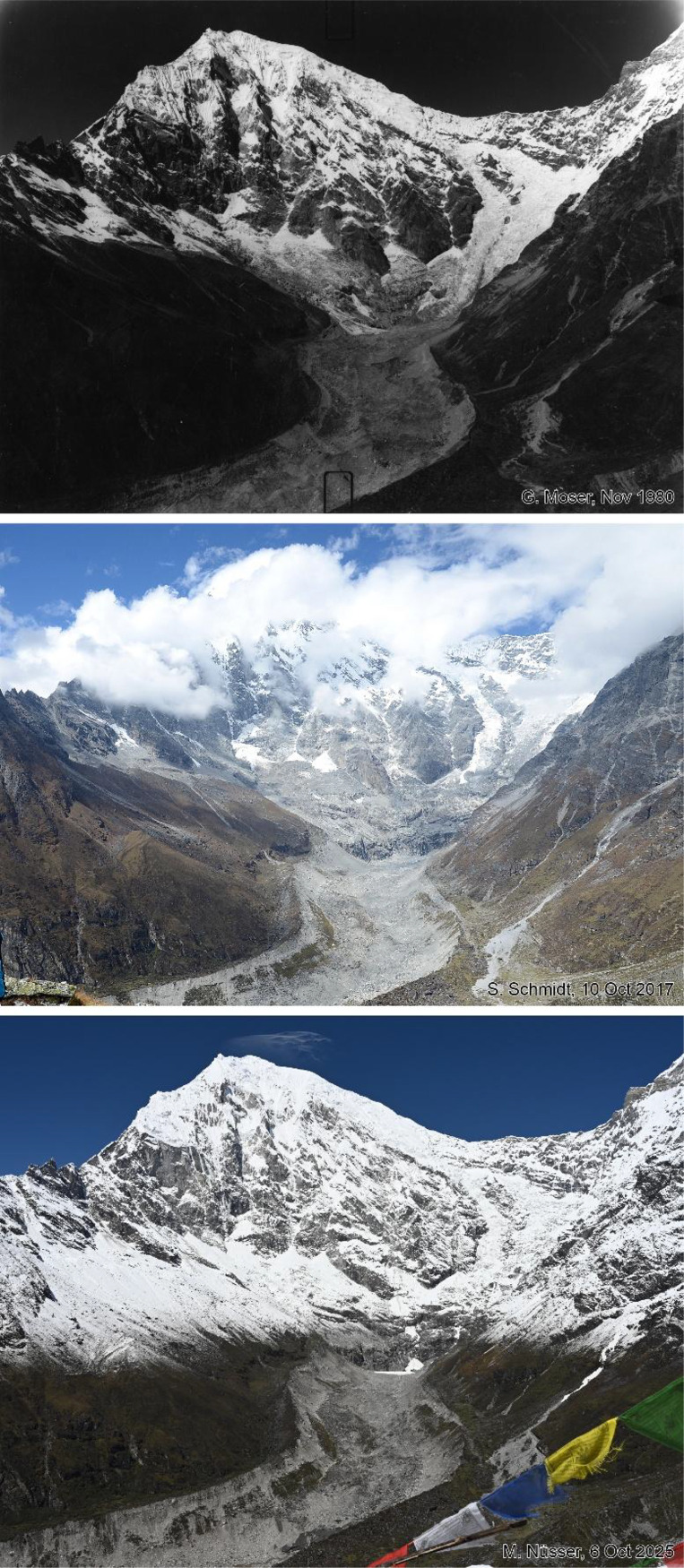
This tri-temporal set of photographs from 1980, 2017 and 2025 shows Langtang Lirung (7234 m) with the debris covered Lirung Glacier in the centre. It was taken from the summit of Kyanjin Ri (4605 m) (viewpoint c). Besides downwasting (thinning) of the debris covered Lirung Glacier, continuous sliding of the inner parts of the lateral moraines can be detected by visual comparison of the photographs. At the foot of the headwall, a snow-filled depression can be detected in the photograph from 2025. The connectivity between glacierised wall and tongue is no longer given. As this photograph was taken after fresh snowfall, no assessment of ice loss of hanging glaciers is possible.

**Fig. 10:**
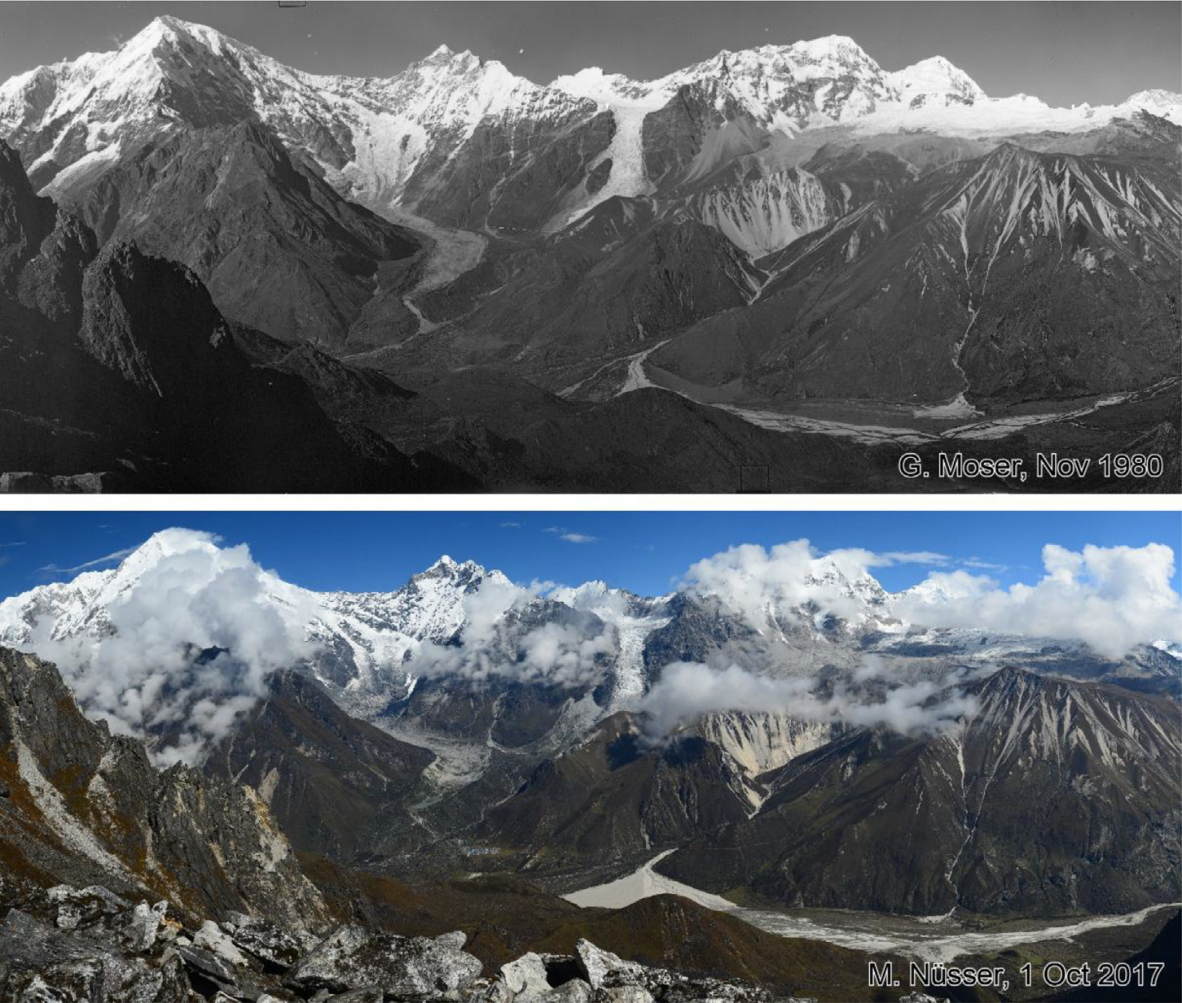
This bi-temporal panorama of matched photographs from 1980 to 2017 shows the south-facing slopes of Langtang Valley between Langtang Lirung (7234 m) on the left-hand side and the summit plateau of Tsergo Ri (5002 m) on the right-hand side. The images were taken from a position at 4800 m a.s.l. near Ganja La (viewpoint d). The debris covered tongue of the Lirung Glacier in the left centre shows downwasting (thinning) and the enlargement of a proglacial lake. The Yala Glacier in the upper right corner of the images shows retreat over the period of 37 years. The alluvial fan at the foot of Tsergo Ri in the centre of both images has been covered by new debris flows in 2017. New landslides occurred on the slope west of Lirung Glacier. The linear erosional structures below Tsergo Ri remain largely unchanged.

**Fig. 11:**
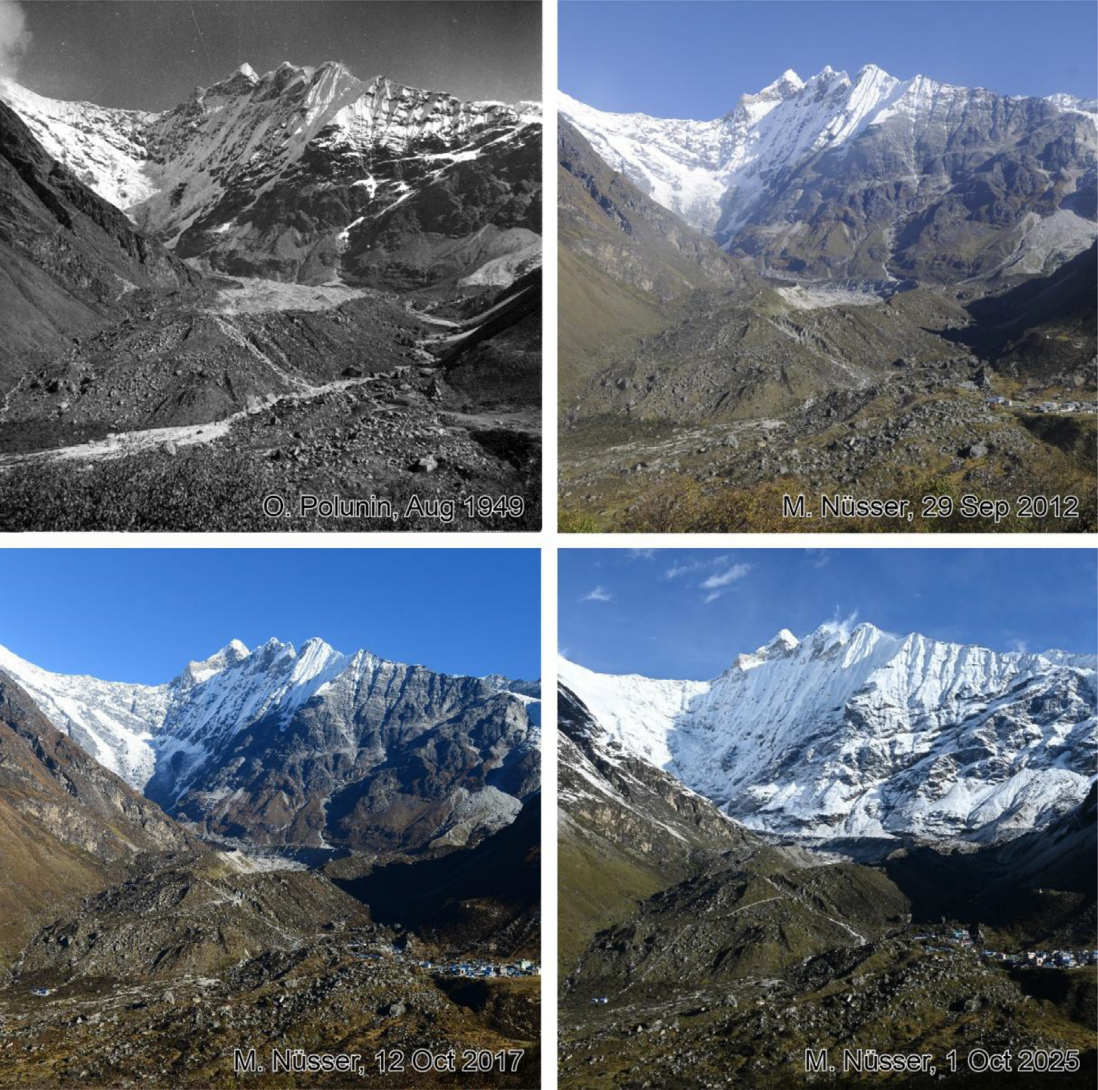
This multi-temporal set of photographs shows the Lirung Glacier with Kimchung (6686 m) in the image centre. The photographs were taken from the north-facing slope of Langtang Valley an altitude of 3940 m (viewpoint e). Massive downwasting (thinning) of the debris covered surface of the Lirung Glacier can be detected. While the south-facing wall is heavily glacierised in the photograph from 1949, significant ice loss can be detected in the images from 2012 to 2017. The photograph from 2025 was taken after fresh snowfall. In the photographs from 2017 to 2025, installations of a microhydro plant for energy production, built after the 2015 earthquake can be identified on the terminal moraine. The settlement Kyanjin Gompa can be detected near the lower right corners in all images (see Fig. 16 for details).

**Fig. 12:**
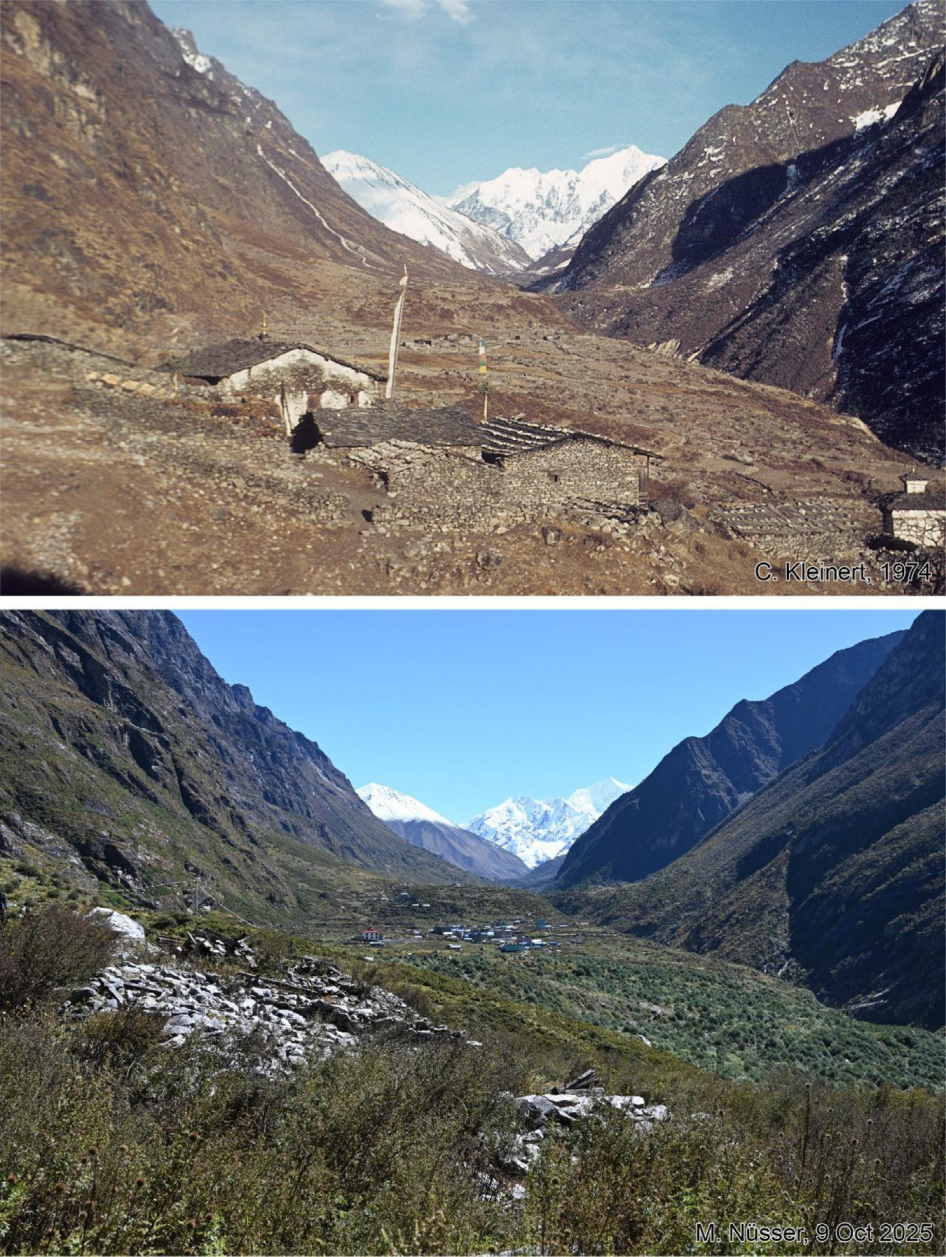
This bi-temporal pair of photographs from 1974 to 2025 shows Langtang Village on the valley floor from viewpoint f with a viewing direction towards east. The stone piles in the replicate photograph are the remains of the 400-years old gompa, destroyed during the 2015 earthquake and visible in the photograph from 1974. An increase in small bush vegetation can be detected on the right-hand side.

**Fig. 13:**
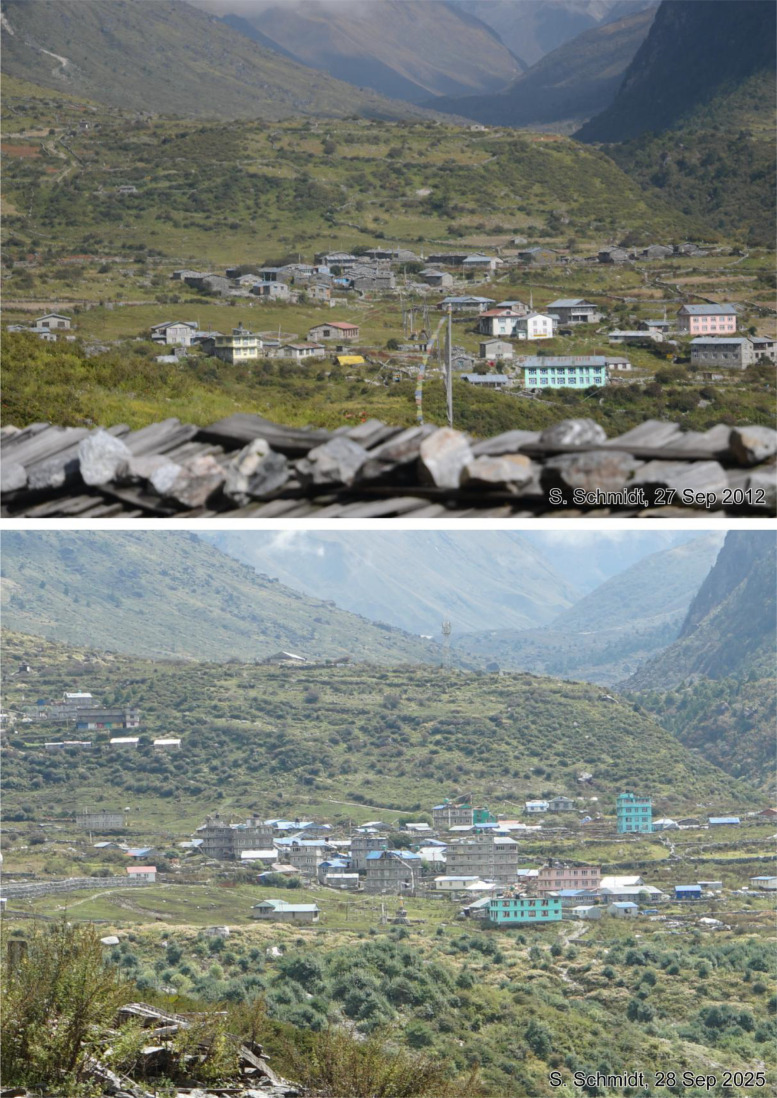
This bi-temporal pair of photographs from 2012 to 2025 shows Langtang Village on the valley floor from viewpoint f with a viewing direction towards east before and after the earthquake. The image from 2012 shows the traditional village with stone houses in the centre and some hotels in the foreground. In the image from 2025, the destroyed village was rebuilt with new hotels. Compared to 2012, most hotels are multi-storey buildings while some of them have a colourful paint. A long gabion structure can be detected on the left-hand side.

**Fig. 14:**
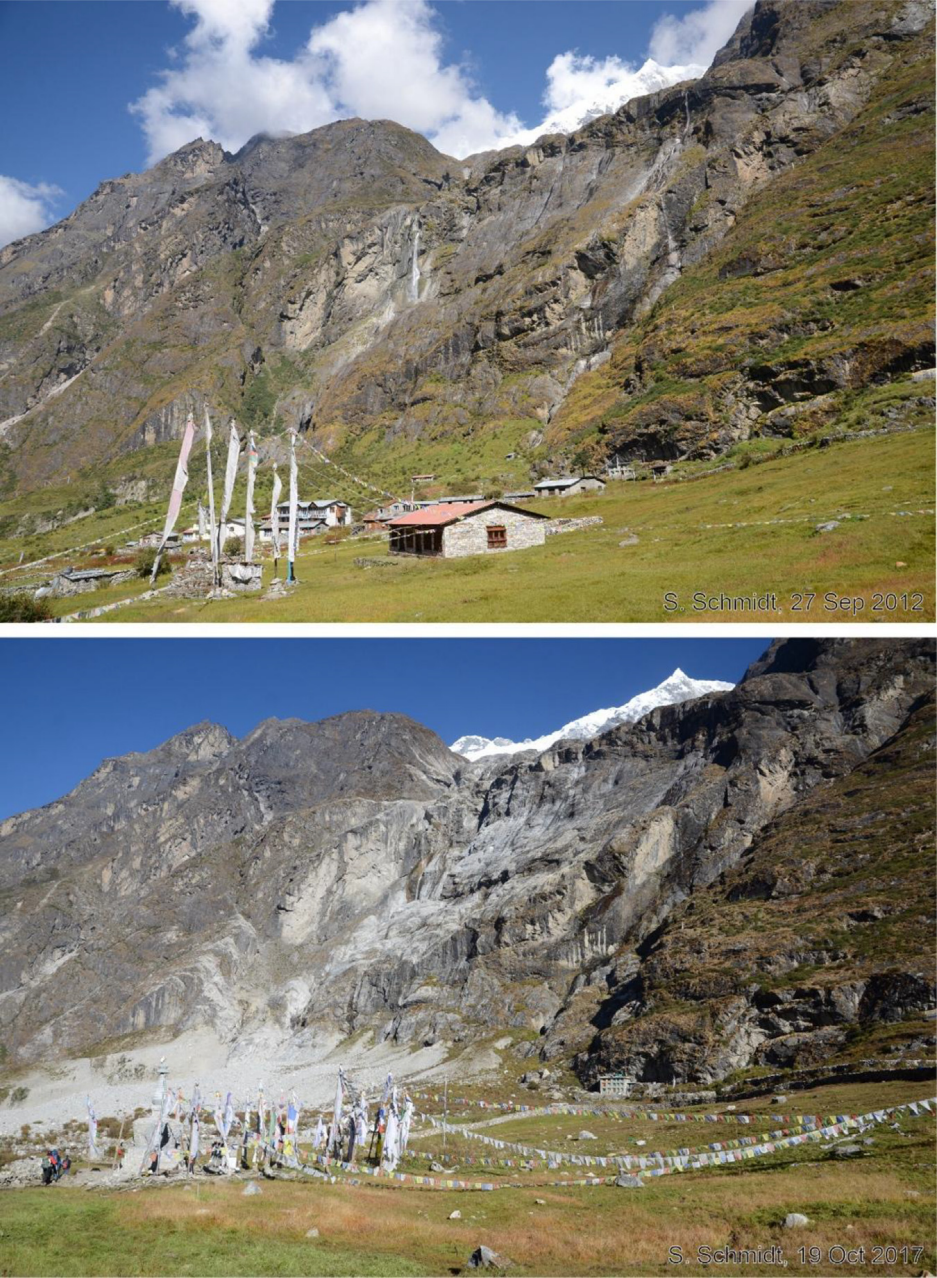
This bi-temporal pair of photographs from 2012 to 2017 shows the northern part of Langtang Village before and after the earthquake. Taken from viewpoint g, the area of the ice and debris avalanche, triggered by the 2015 earthquake is visible behind the memorial place in the image from 2017. Even in 2012, the steep rock slope was almost free of vegetation.

**Fig. 15:**
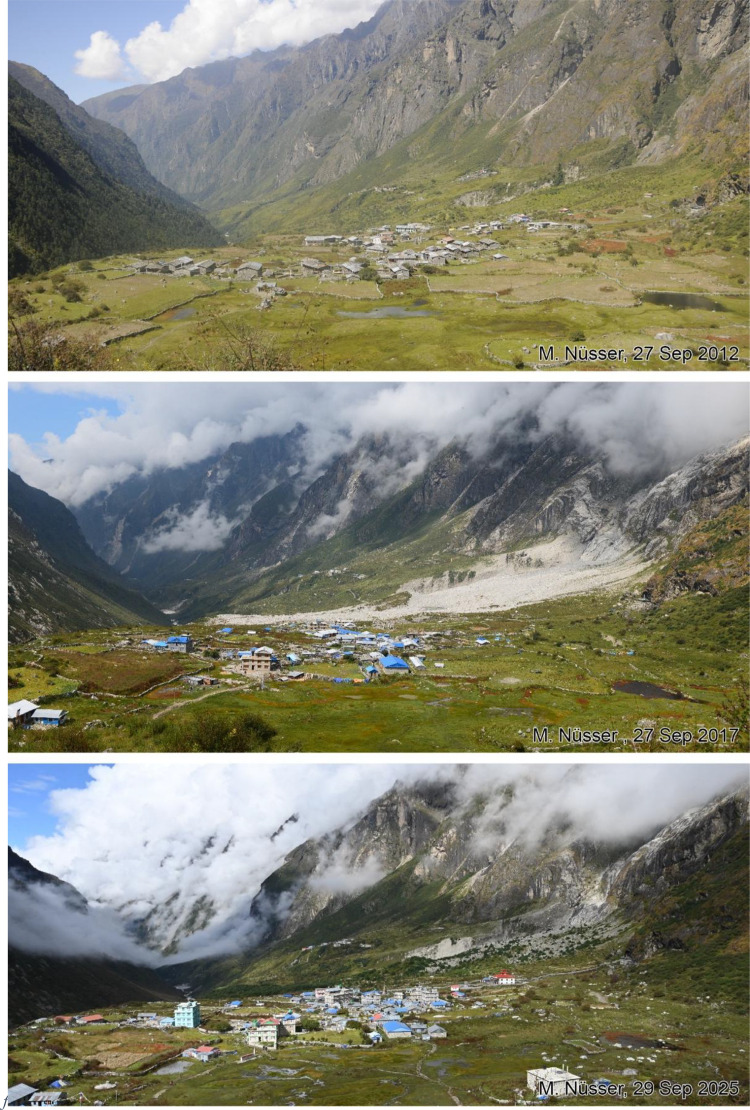
The tri-temporal series of photographs shows Langtang Village with a viewing direction towards east (viewpoint h). It visualises the massive debris cover of the ice and debris avalanche in the background of the 2017 image, which destroyed the village. The former village consisted mainly of traditional stone houses in 2012. The reconstruction after the 2015 earthquake led to a densification and expansion of the village during the urbanisation process, visible in the 2017 and 2025 images. Most agricultural fields and hay meadows are surrounded by small walls.

**Fig. 16:**
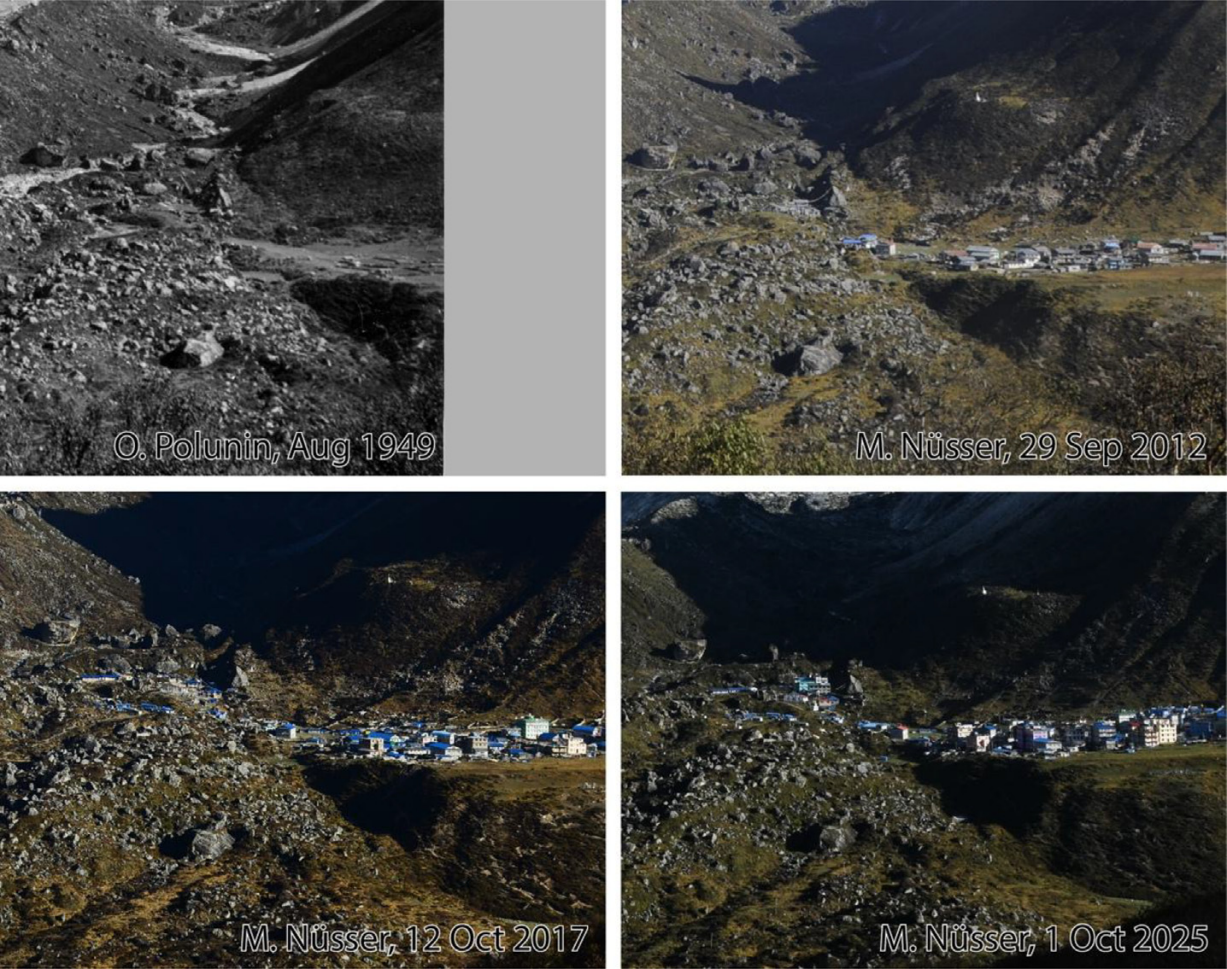
Multi-temporal imagery showing the rapid expansion of Kyanjin Gompa because of the functional change from a seasonal grazing settlement with a few stone huts in 1949 to a booming tourist destination with multi-storey hotels, teahouses and lodges over the last decade. The enlargements of photographs were taken from the north-facing slope of Langtang Valley an altitude of 3940 m (viewpoint e, see Fig. 11).

**Fig. 17:**
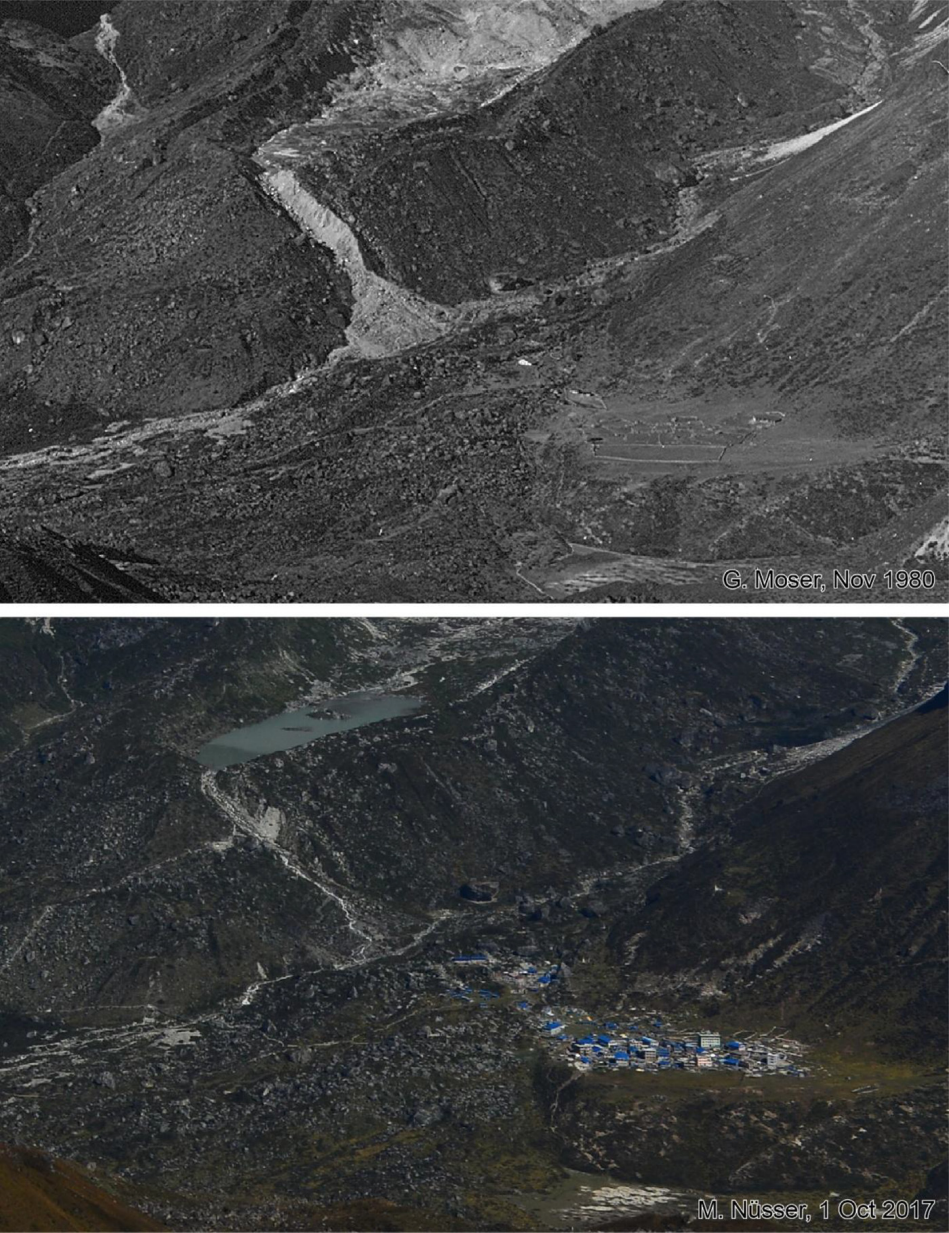
This bi-temporal pair of photographs from 1980 to 2017 shows the development of Kyanjin Gompa from a small hamlet with a few stone huts, animal shelters and agricultural fields surrounded by small walls to a booming tourist destination with many hotels, teahouses and lodges (viewpoint d). An increase of the proglacial lake can be detected. These enlargements of photographs were taken from the north-facing slope of Langtang Valley at an altitude of 4800 m near Ganja La (viewpoint d, see Fig. 10).

**Fig. 18:**
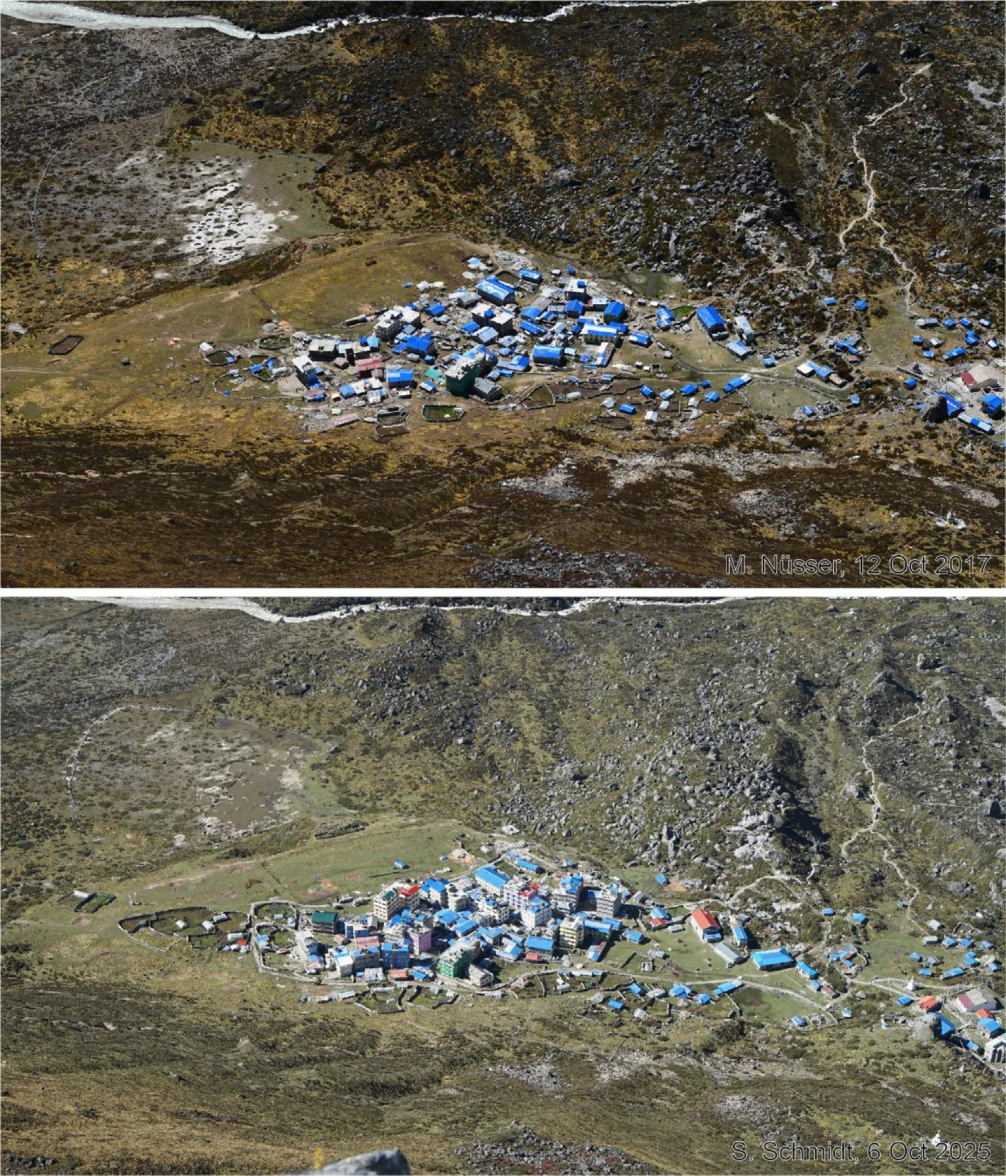
This bi-temporal pair of photographs from 2017 to 2025 shows the most recent development of Kyanjin Gompa after the earthquake (viewpoint i) A densification of the settlement and an increase in the number of storeys on different buildings, exclusively used as hotel, can be detected in the 2025 photograph. The number of greenhouses and vegetable fields have increased, visible on the left-hand side. A stabilised foot path surrounds the village.

**Fig. 19:**
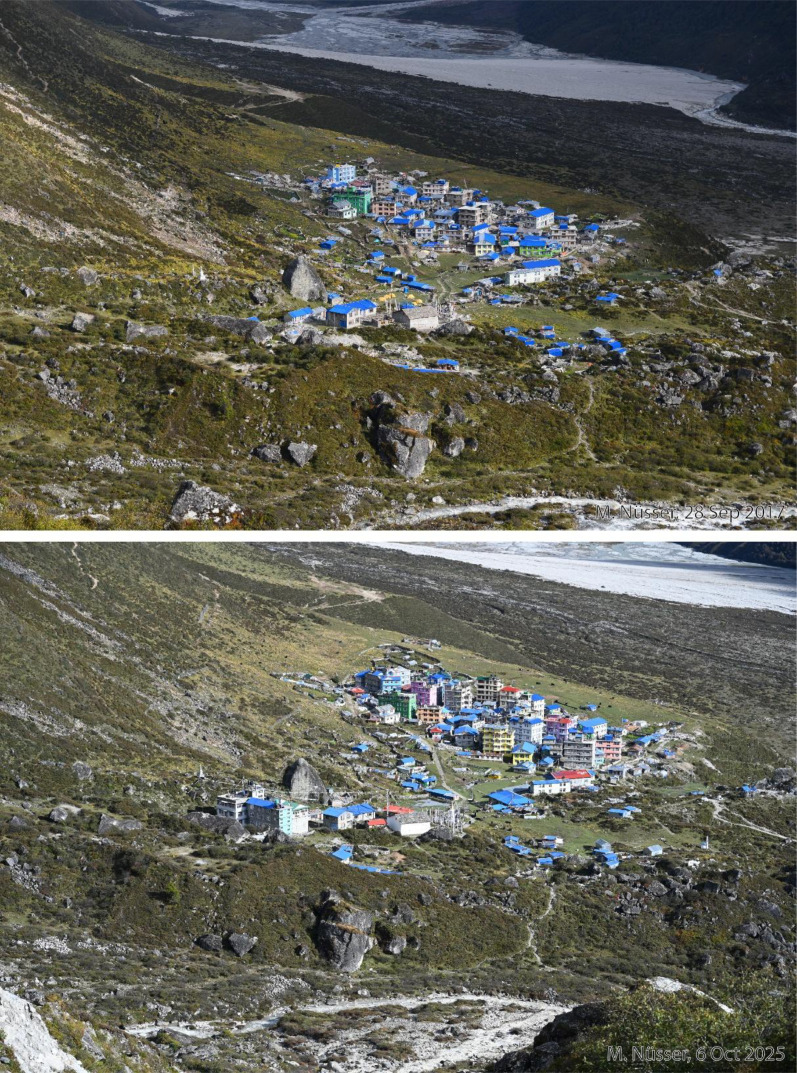
This bi-temporal pair of photographs from 2017 to 2025 shows the most recent development of Kyanjin Gompa after the earthquake from the lateral moraine of Lirung Glacier (viewpoint j). An increase in the number of storeys on different buildings, exclusively used as hotel, and a new hotel on the left (northern) margin of the village can be detected in the 2025 photograph.

**Fig. 20:**
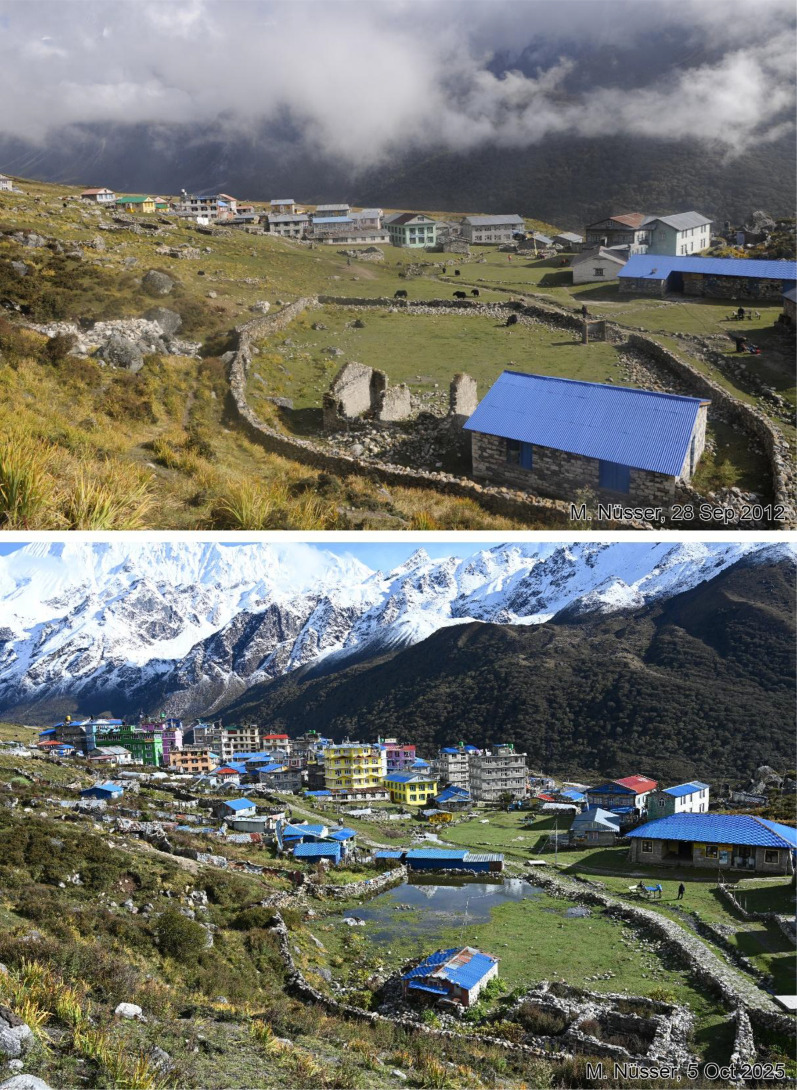
This bi-temporal pair of photographs from 2012 to 2025 shows the development of Kyanjin Gompa before and after the earthquake (viewpoint k). While almost all hotels and guesthouses had only two storeys in 2012, the number has mostly increased to four storeys and additional rooftop terraces.

**Fig. 21:**
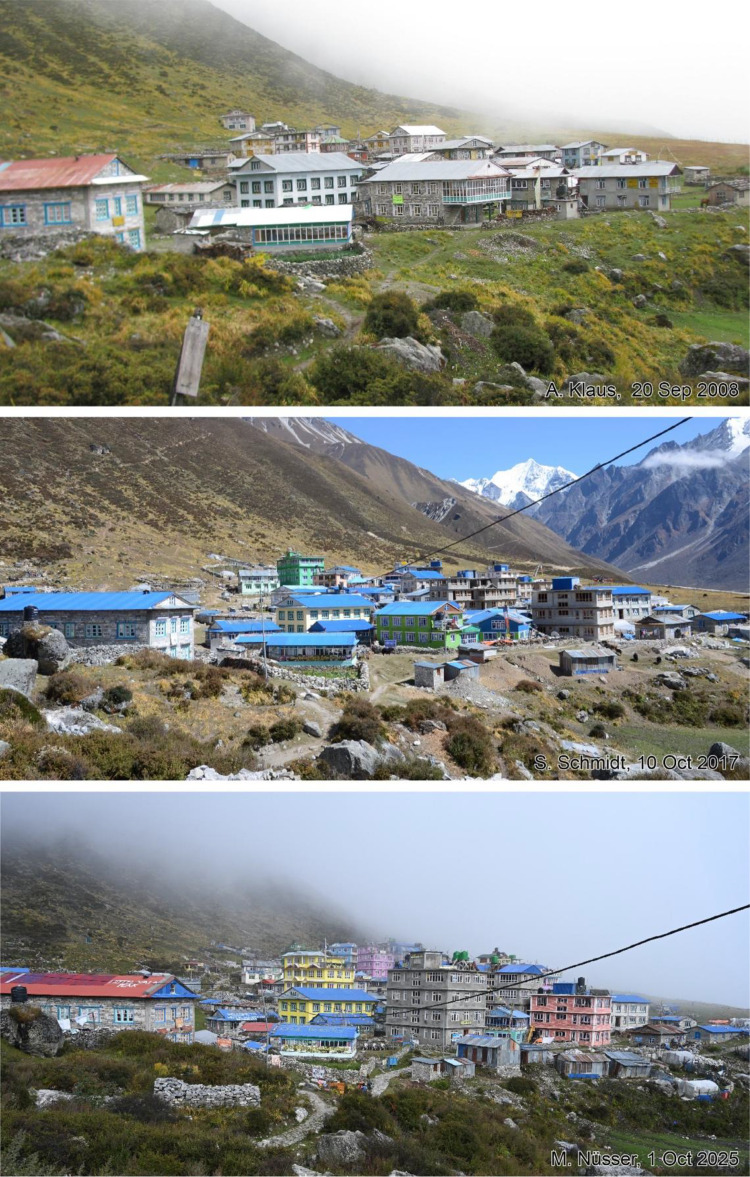
This tri-temporal set of photographs from 2008, 2017 and 2025 shows the development of Kyanjin Gompa before and after the earthquake (viewpoint l). While almost all hotels and guesthouses had only one or two storeys in 2008 and were built in traditional style, the number of storeys has increased to four with additional rooftop terraces. After reconstruction and vertical building extension, some facades received colourful paints.

**Fig. 22:**
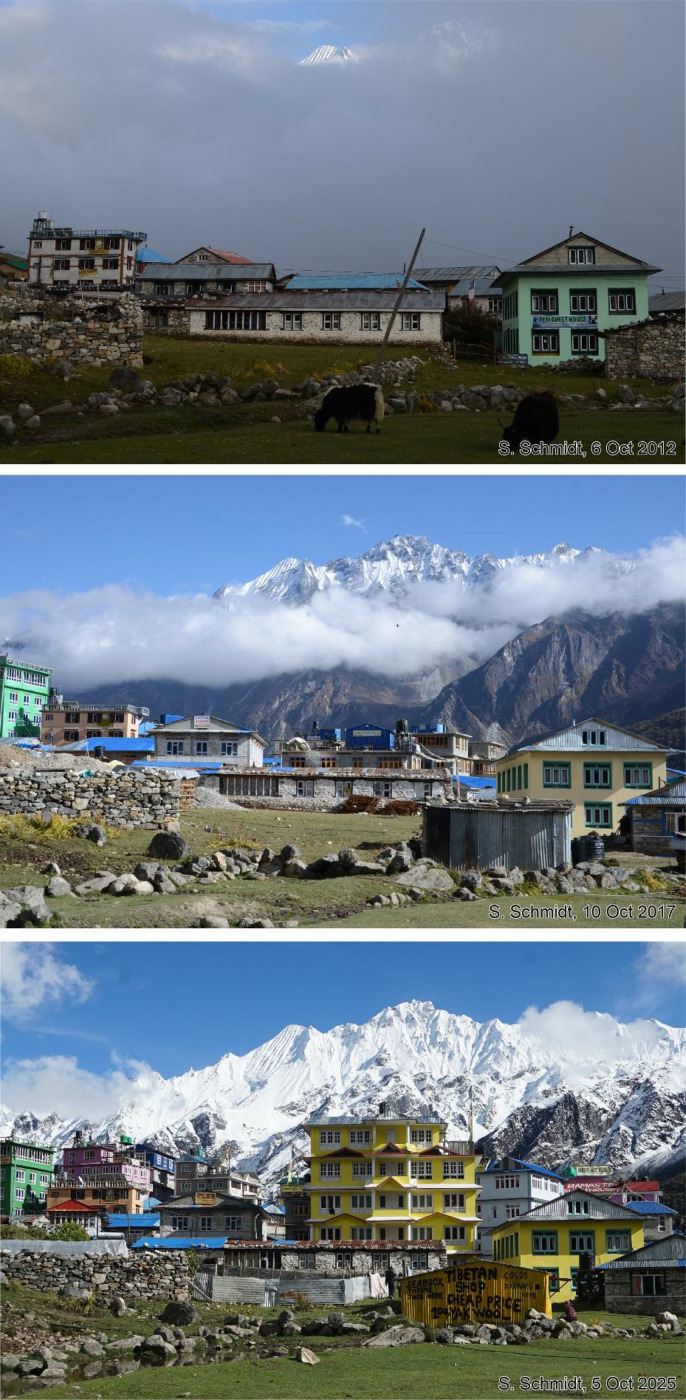
This tri-temporal set of photographs from 2012, 2017 and 2025 shows the development of Kyanjin Gompa before and after the earthquake (viewpoint m). While almost all hotels and guesthouses had only two storeys in 2012, the number has increased to four storeys and additional rooftop terraces. The guesthouse on the right-hand side has not changed.

**Fig. 23:**
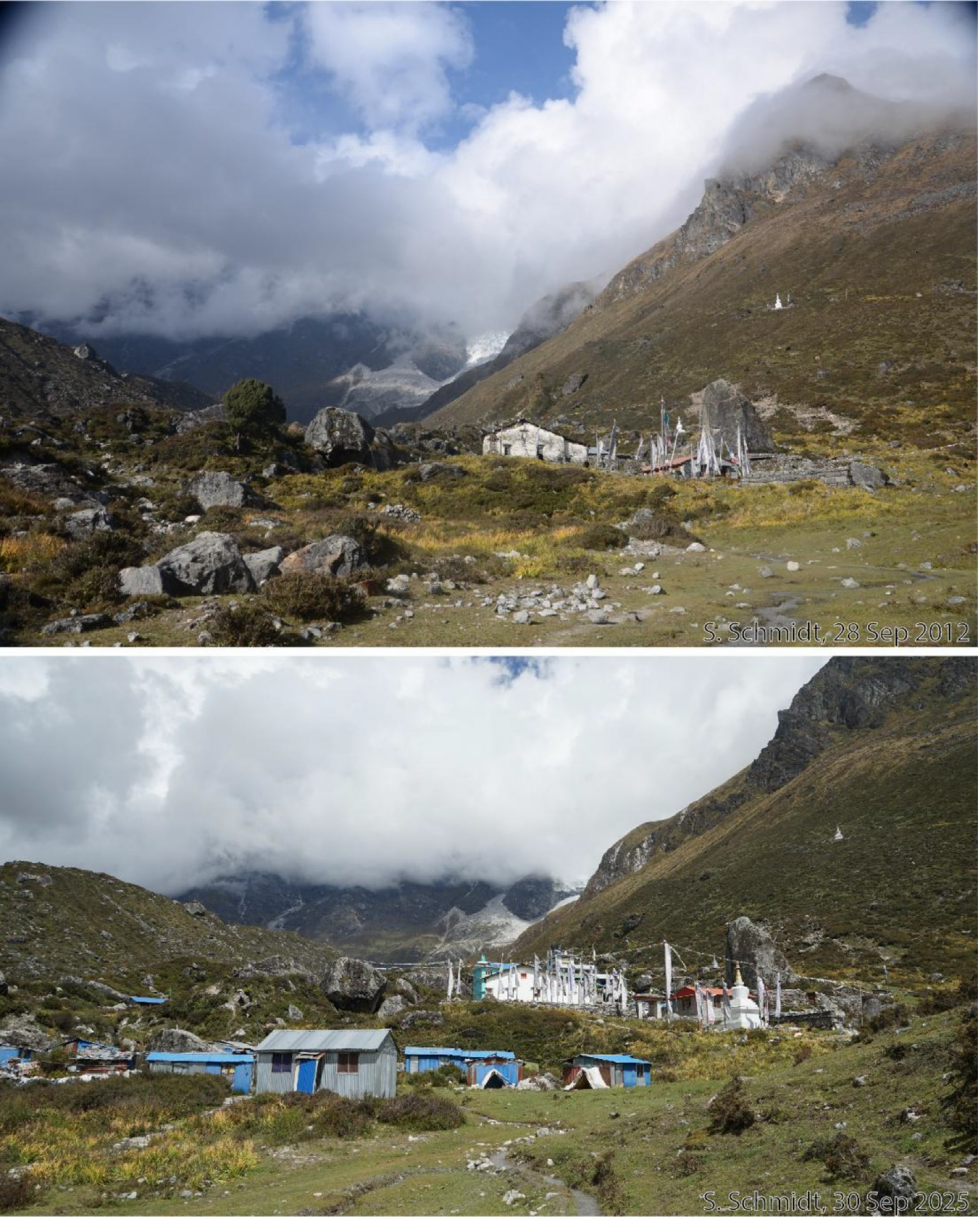
This bi-temporal set of photographs from 2012 and 2025 shows the monastery of Kyanjin Gompa before and after the earthquake (viewpoint n). Temporary shelters still exist from the post-disaster reconstruction phase.

**Fig. 24:**
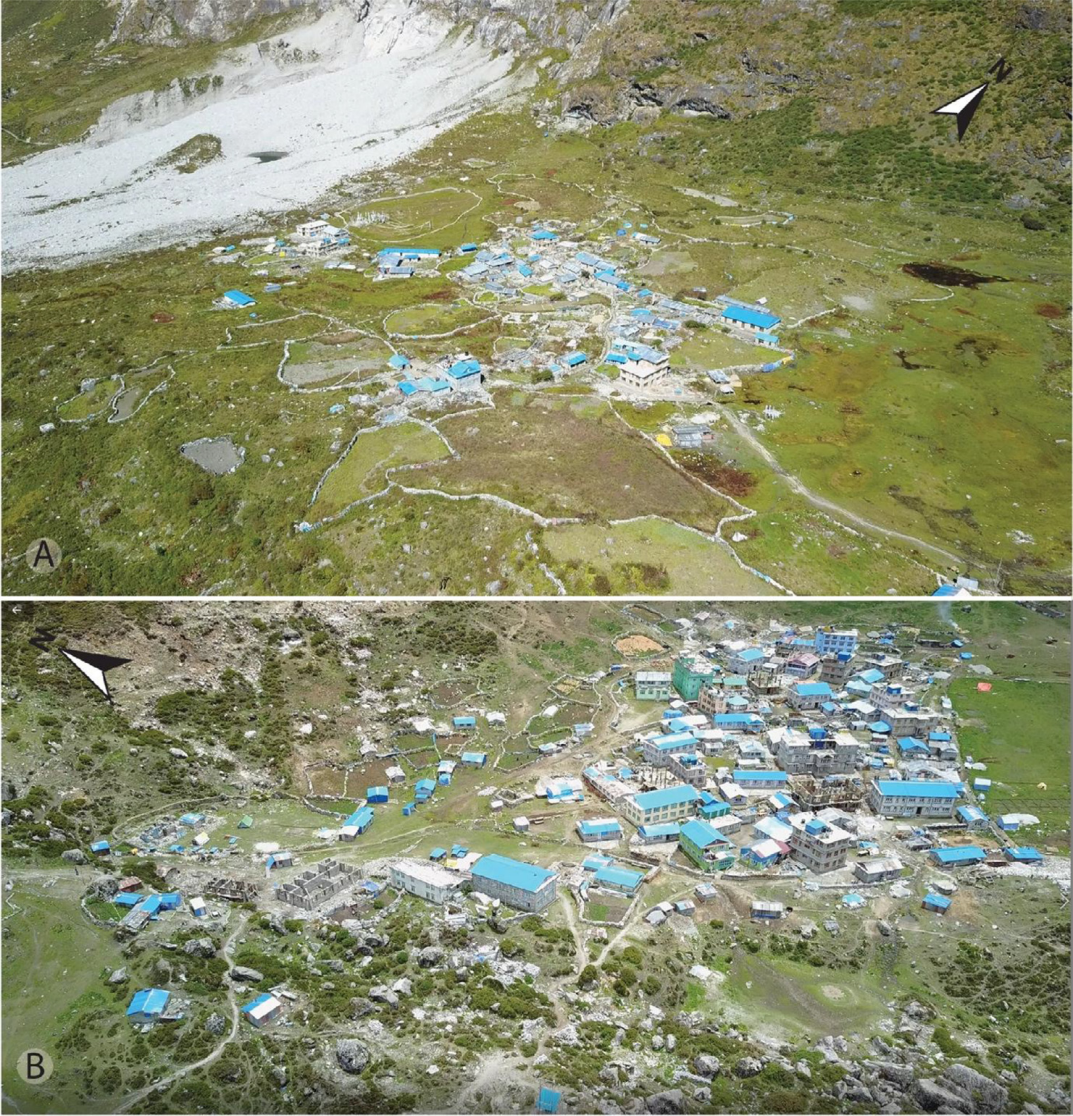
Drone images of Langtang Village from 9 October 2017 (A) and Kyanjin Gompa from 10 June 2018 (B). Most buildings are used for touristic purposes. The blue corrugated iron roofs were built on most buildings after the 2015 earthquake.

This dataset contains multi-temporal photographs from the Langtang Valley in the central Himalaya of Nepal. The historical material includes a set of metric photographs taken by a phototheodolite in 1980, images from 1949 to 1974, combined with a set of matched photographs taken with digital single lens reflex (DSLR) cameras in 2008, 2012, 2017, 2018 and 2025 (Table 1). The historical photographs, originally taken during exploratory expeditions and scientific surveys were collected in various archives and repeated during re-photographic surveys from the same viewpoints. The multi-temporal photographic dataset includes 22 matched images from different sections of the Langtang Valley, with additional two drone images from the main villages of Langtang and Kyanjin Gompa. All camera positions (viewpoints) and viewing directions are marked on maps (Fig. 1). The elevation of the camera positions ranges from 3440 to 5000 m a.s.l. Their locations, including GPS (Global Positioning System) data are listed in Table 2. The photographs are structured in two thematic sets: landscape and glacier images (A), including ten examples from upper Langtang Valley (Fig. 2, Fig. 3, Fig. 4, Fig. 5, Fig. 6, Fig. 7, Fig. 8, Fig. 9, Fig. 10, Fig. 11) and settlement images (B), including twelve examples from Langtang Village and Kyanjin Gompa (Fig. 12, Fig. 13, Fig. 14, Fig. 15, Fig. 16, Fig. 17, Fig. 18, Fig. 19, Fig. 20, Fig. 21, Fig. 22, Fig. 23, Fig. 24).

**Table 1:** Historical and repeat photographs.

**Year**	**Photographer**	**Material**	**Camera, zoom lenses**
1949	Oleg Polunin^1^	Photographs	unknown
1974	Christian Kleinert^2^	Photographs	unknown
1980	Gerhard Moser^3^	Metric Photographs	Phototheodolite, unknown
2008	Alexander Klaus^4^	Photographs	Canon PowerShot SX100 IS, 6 mm
2012	Marcus Nüsser^4^ Susanne Schmidt^4^	Photographs Photographs	NIKON D300S, 28–70 mm NIKON D7000, 16–85 mm
2017	Marcus Nüsser^4^ Marcus Nüsser^4^ Susanne Schmidt^4^	Photographs Drone Photographs	NIKON D800E, 28–70 mm DJI FC220 NIKON D7000, 16–85 mm
2018	Marcus Nüsser^4^ Marcus Nüsser^4^	Photographs Drone	NIKON D800E, 28–70 mm DJI FC220
2025	Marcus Nüsser^4^ Susanne Schmidt^4^	Photographs Photographs	NIKON Z 7.2, 24–70 mm SONY ALPHA II, 24–70 mm

Complete source and archive of analogue and digital data:.

^1^Collection Georg Miehe, Marburg University.

^2^Collection Christian Kleinert, archived at South Asia Institute (SAI), Heidelberg University.

^3^photographic archives of the Austrian Alpine Club (ÖAV), Innsbruck.

^4^Own collections of authors, archived at South Asia Institute (SAI), Heidelberg University.

**Table 2:** Camera locations of photographs used in this study.

**Camera location**	**Viewpoint**	**Figure**	**Lat/Lon**	**Elevation [a.s.l]**
Morimoto Base Camp	a	2–6	85,695 E 28,241 N	4725 m
Tsergo Ri	b	7, 8	85,600 E 28,213 N	5002 m
Kyanjin Ri	c	9	85,576 E 28,223 N	4605 m
Near Ganja La	d	10, 17	85,572 E 28,176 N	4800 m
Near Ngegang Kharka	e	11, 16	85,565 E 28,202 N	3940 m
Langtang Village West	f	12, 13	85,498 E 28,217 N	3440 m
Langtang Memorial	g	14	85,507 E 28,216 N	3450 m
Langtang Village East	h	15	85,513 E 28,216 N	3520 m
Kyanjin Gompa North	i	18	85,571 E 28,213 N	4005 m
Lirung Moraine	j	19	85,563 E 28,219 N	4010 m
Kyanjin Gompa West	k	20	85,566 E 28,214 N	3880 m
Kyanjin Gompa Southwest	l	21	85,565 E 28,213 N	3870 m
Kyanjin Gompa Center	m	22	85,566 E 28,213 N	3860 m
Kyanjin Gompa Monastery	n	23	85,566 E 28,214 N	3870 m

Source: Coordinates and altitudes according to GPS (Global Positioning System) readings during field survey.

### 3.1 Part A: matched landscape and glacier photographs

### 3.2 Part B: matched photographs of settlements and buildings

This collection of photographs focuses on the development of settlement structures in Langtang Village and Kyanjin Gompa: These two largest settlements in the valley are characterized by rapid transformation, mainly driven by mountain tourism. Langtang Village was almost entirely destroyed by a rock-ice avalanche in 2015, triggered by the Nepal earthquake. The second village, Kyanjin Gompa, formerly only a seasonal inhabited settlement for summer grazing, was less severely affected by the earthquake. After the disaster, both villages are characterized by dynamic reconstruction processes, highlighting the importance of tourism and mountain urbanisation. The set of repeat photography collated in this section illustrates these rapid changes in settlement and building structures and functions before and after the earthquake. All viewpoints and viewing directions are marked on maps (Fig. 1) and their GPS data are listed in Table 2.

## 4 Experimental Design, Materials and Methods

Repeat photography is the act of taking bi- or multi-temporal replicates of photographs to produce images that match the original ones from identical viewpoints. Comparisons of the original photographs and their replicates allow for assessments of change or persistence of landscape elements. Frequently practiced in the form of re-photographic surveys, this method has evolved as a valuable tool in mountain research to detect and visualize changes in glaciers [[2], [3], [4], [5]] as well as in socio-ecological topics such as settlement and land use patterns [6,7].

The selection of historical data from Langtang Valley includes one image from the first western expedition to Nepal in 1949 [8,9], one image from 1974 with a focus on settlement and building structures [10,11], and eight metric photographs from 1980, that were taken for topographical mapping [12]. Historical images were repeated during field surveys in 2012, 2017, 2018, and 2025. Some of the historical photographs were also used for detailed regional vegetation surveys and assessments of natural history [13,14]. To document changes in settlement structure and buildings in Langtang Village and Kyanjin Gompa before and after the 2015 Nepal earthquake, the method of repeat photography was used with material from our own collections, including drone images from 2017 to 2018 that provide an overview of post-disaster reconstruction activities.

Due to its large temporal and spatial coverage, the dataset provides a multi-scale baseline for a better understanding of landscape, glacier and settlement changes in the Langtang Valley. The dataset provides reference data for a co-submitted article [1] with original archival material and repeat images [15].

## Limitations

The method of repeat photography depends on the availability of original photographic material from a region that meets the requirements of visual quality and thematic relevance. Bad weather conditions, shadowing effects from surrounding topography or from clouds can impede visual comparison between photographs in sufficient detail. Seasonal effects of snow cover distribution and phenology are further potential limitations.

## Ethics Statement

The authors have read and follow the ethical requirements for publication in Data in Brief and confirm that the current work does not involve human subjects, animal experiments, or any data collected from social media platforms.

## CRediT Author Statement

**Marcus Nüsser:** Conceptualization, Investigation, Data Curation, Writing – Original Draft, Reviewing and Editing, Supervision. **Susanne Schmidt**: Data Curation, Investigation, Writing - Original Draft. **Alexander Klaus**: Visualization, Investigation, Writing – Original Draft.

## Data Availability

Mendeley DataMulti-temporal photographic dataset of landscape changes in Langtang Valley, Nepal (Original data).
